# Beyond the cut-off: harmonizing kappa free light chain index using likelihood ratios improves the clinical interpretation in the diagnosis of multiple sclerosis

**DOI:** 10.3389/fimmu.2025.1672662

**Published:** 2025-12-03

**Authors:** Matthijs Oyaert, Louis Nevejan, Cathérine Dekeyser, Pieter De Kesel, Melissa Cambron, Ludo Vanopdenbosch, Liesbeth Van Hijfte, Guy Laureys, Martine Vercammen

**Affiliations:** 1 Department of Laboratory Medicine, Universitair Ziekenhuis Gent, Ghent, Belgium; 2 Department of Laboratory Medicine, Algemeen Ziekenhuis Sint-Jan Brugge AV, Bruges, Belgium; 3 Department of Microbiology, Immunology and Transplantation, Clinical and Diagnostic Immunology, Katholieke Universiteit Leuven, Leuven, Belgium; 4 Department of Neurology, Universitair Ziekenhuis Gent, Ghent, Belgium; 5 Department of Laboratory Medicine, Algemeen Ziekenhuis Maria Middelares, Ghent, Belgium; 6 Department of Neurology, Algemeen Ziekenhuis Sint-Jan Brugge AV, Bruges, Belgium; 7 Faculty of Medicine and Health Sciences, Universiteit Gent, Ghent, Belgium; 8 Basic Biomedical Sciences, Vrije Universiteit Brussel, Brussels, Belgium

**Keywords:** Kappa free light chain index, likelihood ratios, multiple sclerosis, Kappa free light chains, Freelite-Optilite versus N Latex-BNII, harmonization

## Introduction

The kappa free light chain (κFLC) index, defined as the quotient of the κFLC ratio to albumin ratio in cerebrospinal fluid (CSF) and serum, was incorporated in the 2024 revisions of the McDonald criteria for multiple sclerosis (MS) diagnosis (1, 2). Recent evidence indicates that the κFLC index tested with validated assays quantifies intrathecal immunoglobulin synthesis with similar diagnostic accuracy for MS as CSF-specific oligoclonal bands (OCBs) (3–5). The 2017 revisions of the McDonald criteria facilitated diagnosis with the inclusion of CSF oligoclonal bands as a substitute for dissemination in time (DIT), with the 2024 revisions adding the κFLC index as interchangeable with oligoclonal bands. However, absolute κFLC values differ between methods, resulting in a method-specific cut-off for the κFLC index (3–8).

The optimal κFLC index cut-off is a matter of debate (1). A laboratory cut-off depends on many variables, such as the patient groups that need to be discriminated, the required sensitivity and specificity, the laboratory reagents, and, to a lesser extent, the analyzer. Clinicians prefer a clear-cut, binary classification of laboratory test results. However, the interpretation often cannot be considered black or white but demands expertise. It is the responsibility of laboratory specialists to support clinicians in making the final interpretation. In autoimmune serology, there is growing recognition of the value of reporting likelihood ratios (LRs) for specific diseases alongside the manufacturer’s cut-off values (9, 10). The LR is defined as the prevalence of patients with a particular test result divided by the prevalence of controls with the same test result. For instance, a test result with a LR of 10 indicates that the test result is 10 times more likely in patients than in controls. Using the LR, the probability of a specific disease can be calculated when the pre-test probability, based on clinical signs and other investigations, is defined. In light of the ongoing debate about the optimal cut-off value of the κFLC index for diagnosing MS, we propose the use of the κFLC index LR in addition to a fixed numerical threshold. This approach allows for a more clinically meaningful interpretation of test results. Moreover, the κFLC index LR enables the comparison of laboratory results obtained using different reagents or analyzers, allowing different numerical values to be assigned the same clinical significance. In clinical practice, the LR concept is the most effective when specific result intervals, rather than individual test values, are associated with a defined LR.

In this study, we demonstrate that using interval-specific LRs allows for the harmonization of method-dependent cut-offs and provides additional clinical value in the diagnosis of MS by enabling the calculation of post-test probabilities (10).

## Methods and results

**Figure 1:**
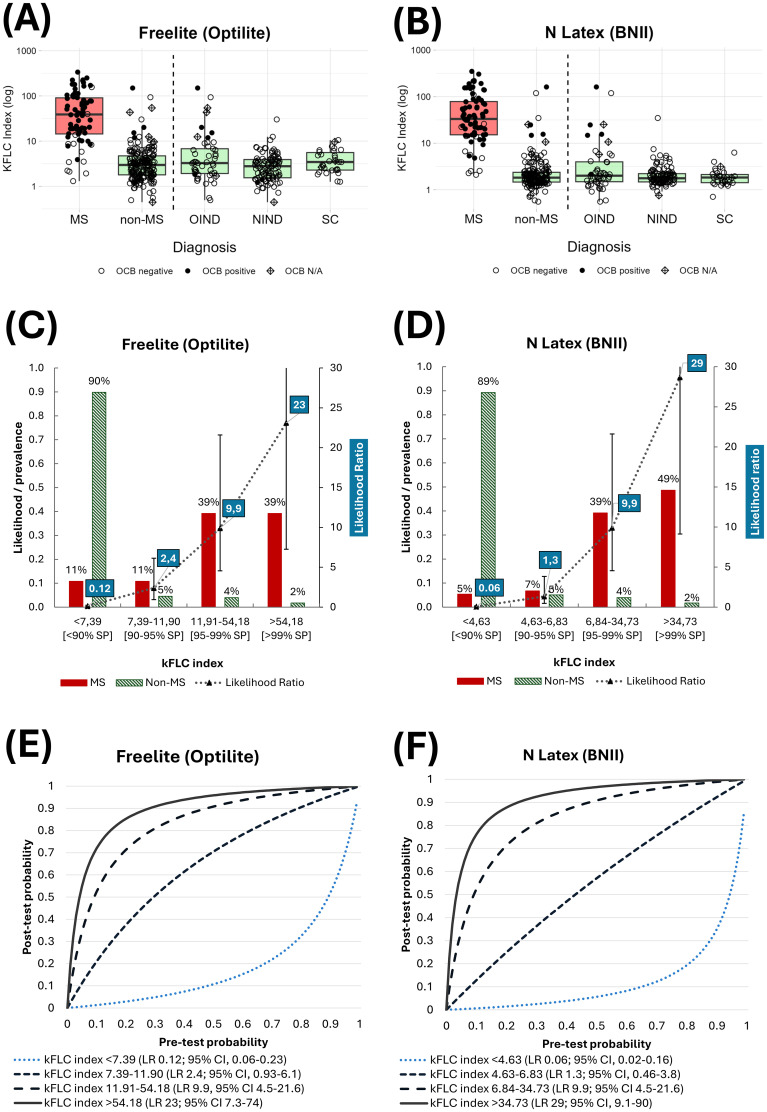
**(A, B)** Boxplots of κFLC index results measured using Freelite^®^**(A)** and N Latex^®^**(B)** in MS and non-MS patients, including OCB test results. **(C, D)** Prevalence (likelihood) of MS and non-MS according to κFLC index test result intervals, which are based on <90%, 90%–95%, 95%–99%, and >99% specificity levels. The corresponding likelihood ratios with 95% confidence intervals to the test result intervals are shown in the second y-axis. **(E, F)** Post-test probability for MS as a function of pre-test probability for different test result intervals (depicted as corresponding likelihood ratios including 95% confidence intervals). OCB, oligoclonal bands; MS, multiple sclerosis; NIND, non-inflammatory neurological disorder; OIND, other inflammatory neurological disorder; SC, symptomatic controls; SP, specificity; κFLC, kappa free light chain.

The LR concept was applied to a previously published patient cohort from two centers (4) using reagents from The Binding Site (Freelite^®^) and Siemens (N Latex^®^) in parallel on the same samples (**Figures 1A, B**). Paired serum and CSF samples from patients with neurological diseases were collected from the AZ Sint-Jan Bruges and UZ Ghent University hospital biobanks. Seventy-four patients were diagnosed with MS using the revised 2017 McDonald criteria, 49 patients suffered from other inflammatory/infectious neurological diseases of the central and peripheral neurological system, and 98 patients suffered from non-inflammatory neurological diseases. In addition, 30 symptomatic controls were included without evidence of organic central or peripheral nervous system disease. κFLC and albumin were measured on Optilite, using the Freelite^®^ Mx κ (kappa) Free assay and Low-Level Albumin assay from The Binding Site (The Binding Site, Birmingham, UK) versus the N Latex^®^ reagent and the N Albumin antiserum anti-human albumin kit on BN II (Siemens Healthineers GmbH, Marburg, Germany). Diagnostic performance characteristics (specificity and LR) were calculated to discriminate MS patients (n = 74) from disease controls (n = 177). Next, test result interval-specific LRs were calculated based on predefined specificity levels <90%, 90%–95%, 95%–99%, and >99%. All analyses were performed using MedCalc Statistical Software version 17.6 (MedCalc Software bvba, Ostend, Belgium) and RStudio version 2024.

Although the nominal values for the κFLC index corresponding to the predefined specificity levels differ between the Freelite^®^ and N Latex^®^ assays, an increased probability of MS was observed in both methods with increasing κFLC index. When the κFLC index was within the 95%–99% specificity cut-off of either assay (11.91–54.18 for Freelite^®^ and 6.84–34.73 for N Latex^®^), the LR for both assays was 9.9 (95% confidence interval, 4.5–21.6), indicating that this test result was **±** 10× more likely to be found in patients with MS at diagnosis compared to patients without MS (**Figures 1C, D**). In contrast, patients were ± 10 times less likely to have MS when the κFLC index was below the 90% specificity cut-off of either assay (<7.39 for Freelite^®^ and <4.63 for N Latex^®^) (**Figures 1C, D**). Of the patients with MS, 39% and 49% had a κFLC index >54.18 (Freelite^®^) and >34.73 (N Latex^®^), respectively. Ninety percent of the controls had a κFLC index <7.39 (Freelite^®^) and <6.8 (N Latex^®^). **Table 1** shows two case examples of how LRs can be integrated into the laboratory report. These LRs can be automatically generated using the laboratory information system (LIS) based on the κFLC index result.

**Table 1:** Two case examples of communicating likelihood ratio values in a clinical laboratory report.

Case 1	
Laboratory A—Freelite^®^ (Optilite)	
Kappa FLC index	9.35
>6.10: positive (Montalban et al., *Lancet Neurol*, 2025;24:850–65)	
Likelihood ratio for multiple sclerosis at diagnosis	2.4 (95% CI, 0.9–6.1)
Laboratory B—N Latex^®^ (BN II)	
Kappa FLC index	5.47
>6.10: positive (Montalban et al., *Lancet Neurol*, 2025;24:850–65)	
Likelihood ratio for multiple sclerosis at diagnosis	1.3 (95% CI, 0.5–3.8)
Case 2	
Laboratory A—Freelite^®^ (Optilite)	
Kappa FLC index	19.56
>6.10: positive (Montalban et al., *Lancet Neurol*, 2025;24:850–65)	
Likelihood ratio for multiple sclerosis at diagnosis	9.9 (95% CI, 4.5–21.6)
Laboratory B—N Latex^®^ (BN II)	
Kappa FLC index	10.65
>6.10: positive (Montalban et al., *Lancet Neurol*, 2025;24:850–65)	
Likelihood ratio for multiple sclerosis at diagnosis	9.9 (95% CI, 4.5–21.6)

In case 1, laboratory A (using the Freelite^®^ assay on Optilite to measure the κFLC index) reported a value of 9.35, which is above the proposed cut-off (6.10) in the 2024 revised McDonald criteria for the diagnosis of multiple sclerosis (MS). For the same sample, laboratory B (using the N Latex^®^ assay on BN II to measure the κFLC index) reported a value of 5.47, which is below the proposed cut-off. When likelihood ratios are reported in addition to the single cut-off, it can be concluded that this particular test result does not help to either support or exclude the diagnosis of MS, as the confidence interval (CI) includes 1. This patient was not diagnosed with MS but with another inflammatory neurological disorder. In case 2, laboratory A reported a κFLC index value of 19.56, which is ±3× above the proposed cut-off (6.10). For the same sample, laboratory B reported a κFLC index of 10.65, which is only ± 1.5× above the proposed cut-off (6.10). Based on the reported likelihood ratios, it can be concluded that the interpretation of the two test results is identical: this particular test result implies a clinically important difference in pre-test–post-test odds (in favor of MS diagnosis). This patient was diagnosed with MS.

By combining the pre-test probability for MS, based on the clinical presentation and MRI criteria for dissemination in space (3), one can calculate the post-test probability for MS. As visualized in **Figures 1E, F**, if the pre-test probability is, for example, 40%, the post-test probability for MS is 87% when a κFLC ratio between 11.9 and 54.2 is obtained using the Freelite^®^ assay (**Figure 1E**) and 87% when a κFLC ratio between 6.8 and 34.7 is obtained using the N Latex^®^ assay (**Figure 1F**). One could also calculate these probabilities based on pre-test and post-test odds (10).

## Discussion

Clinical laboratories should consider reporting interval-specific LRs alongside numerical cut-offs. This is especially important for immunological tests like the κFLC index, where results differ across platforms due to a lack of standardization. As demonstrated in this study, a high positive LR (ideally >10) is the most effective for confirming a diagnosis of MS, while a low negative LR (ideally <0.1) is the most useful for ruling it out. A LR > 10 does not mean 100% specificity for MS. Very high κFLC index ratios can also occur in non-MS patients, such as other inflammatory disorders of the nervous system, central nervous system infections, or monoclonal protein infiltration. The addition of test result interval-specific LRs next to a single cut-off on the laboratory report adds value since it is more informative than just a dichotomous interpretation based on sensitivity and specificity (i.e., a positive test result suggests the presence of disease, while a negative test result indicates the absence of disease (10)). As shown in **Table 1**, LRs can be automatically calculated using the LIS based on the κFLC result and included in the laboratory report next to the single cut-off. The examples in **Table 1**, which are actual samples used in this study, highlight that the quantitative values between Freelite^®^ and N Latex^®^ can differ, causing a different interpretation when a single cut-off is used. However, when LRs are added to the laboratory report, the interpretation of the two assays is harmonized, providing additional clinical information. It can be useful to add a non-technical explanation regarding the interpretation of the LRs (e.g., for a test result with a LR of 9.9, “the test result is 9.9 times more likely to be found in individuals with multiple sclerosis at diagnosis than in individuals without multiple sclerosis”).

Knowledge of pre-test probability is not required to calculate test result interval-specific LRs, but if available, it can be useful to compute the post-test probability of disease. If a clinician adds the pre-test probability information when requesting a κFLC index analysis, the LIS can automatically calculate and report the post-test probability for MS. The report could display the quantitative κFLC index, the single cut-off, the LR, and the post-test probability for MS, accompanied by a comprehensive conclusion. For example, if the pre-test probability of the second case in **Table 1** is 50%, the automatically generated conclusion could state, “Estimated probability of multiple sclerosis is 91% given a pre-test probability of 50%”. The graphs provided in **Figures 1E, F** could accompany the report to visualize the interpretation of the LR. However, follow-up studies are needed for this to convert clinical data into pre-test probability percentages.

The calculation of test result interval-specific LRs requires a multicenter approach to ensure the inclusion of a sufficient number of patient and control samples. The composition of these groups can be tailored to the specific clinical question and the populations that need to be distinguished.

To conclude, interval-specific LRs informs clinicians about the likelihood of disease, independent of the laboratory platform or assay used, and offers a practical way to standardize test outcomes. This allows a more unambiguous interpretation of test results (10). An important role of the laboratory specialists is to educate clinicians about the added value of interval-specific LRs delivered by the laboratory.
